# Allergic Bronchopulmonary Aspergillosis With or Without Asthmatic Symptoms?

**DOI:** 10.7759/cureus.15498

**Published:** 2021-06-07

**Authors:** Victor Acosta-Rivera, Jesus M Melendez-Montañez, Wilfredo De Jesús-Rojas

**Affiliations:** 1 Medicine, Ponce Health Sciences University, Ponce, PRI; 2 Biology, University of Puerto Rico, Mayagüez Campus, Mayagüez, PRI; 3 Pediatrics, Ponce Health Science University, Ponce, PRI; 4 Pediatrics, University of Puerto Rico, Medical Sciences Campus, San Juan, PRI

**Keywords:** allergic broncho-pulmonary aspergillosis, asthma, non-cf bronchiectasis, rare lung diseases, aspergillus fumigatus

## Abstract

Allergic bronchopulmonary aspergillosis (ABPA) is a localized inflammatory airway disease seen in patients sensitized to *Aspergillus fumigatus (A. fumigatus) *antigens. The disease presents with productive cough, wheezing, episodic fever, as well as central bronchiectasis (CB) and mucus plugs on computed tomography (CT) scans. If treated accordingly, symptoms and pulmonary damage caused by ABPA can be reverted. Currently, the diagnostic criteria for ABPA require the diagnosis of predisposing pulmonary diseases such as asthma and cystic fibrosis (CF) in order to establish the diagnosis. There has been an increasing number of cases reporting ABPA without evidence of past asthmatic history or symptoms. This reflects the need for more sensitive diagnostic tests in order to prevent progression to irreversible lung injury. Here we report a 22-year-old Puerto Rican male who went undiagnosed for ABPA for 12 months due to the absence of asthma or CF history.

## Introduction

*Aspergillus fumigatus (A. fumigatus)* is a fungus found ubiquitously throughout the environment and induces a localized hypersensitivity reaction of the lower airways. This disease, known as allergic bronchopulmonary aspergillosis (ABPA), typically manifests in patients who have aspergillus sensitization (AS), as well as those who suffer from other chronic pulmonary diseases including asthma and cystic fibrosis (CF). ABPA presents as an acute or subacute clinical deterioration from baseline with increased cough, sputum production or sputum color change, wheezing, dyspnea, as well as constitutional symptoms such as weight loss and fever, that fails to respond to appropriate medical therapy [1].

There are multiple diagnostic criteria, which include a combination of radiological findings as well as serologic and immunologic studies, in order to establish the diagnosis of ABPA. The vast majority of them either include the presence of asthma or CF as an integral part of the diagnostic algorithm, or the criteria is specifically made for patients with CF [2, 3]. However, an increasing number of reported ABPA cases do not strictly satisfy all diagnostic criteria or are presented atypically, which causes delays in diagnosis and subsequent initiation of appropriate medical treatment [4-8]. ABPA has been shown to revert pulmonary pathology when treated appropriately and in a timely manner. Conversely, unrecognized and untreated ABPA may lead to irreversible lung damage and increased overall morbidity and mortality [9]. In this report, we discuss the case of a 22-year-old Puerto Rican male patient who went underdiagnosed and undertreated for ABPA due to the lack of previous asthma or CF history. 

## Case presentation

A 22-year-old non-smoker Puerto Rican male presented to our services with a 12-month history of shortness of breath, cough productive of thick, brown, well-formed sputum, chest pain, episodic fevers and general malaise. Symptoms occurred acutely with no identifiable precipitating factors. Past medical history prior to the development of his symptomology was unremarkable. Family history was negative for pulmonary disease or similar symptoms in the household. Over this time period, he required seven hospitalizations for presumed recurrent mycoplasma pneumonia. Physical examination revealed bilateral polyphonic wheezes on auscultation that were appreciated on all lung fields. Spirometry demonstrated severe airway obstruction with a forced vital capacity (FVC) of 43%, forced expired volume in one second (FEV1) of 26%, and FEV1/FVC of 62% predicted values with a 20% of predicted change in FEV1 on the post-bronchodilator test. Fractional excretion of nitric oxide (FeNO) was 105 ppm (normal < 30 ppm). 

**Figure 1:**
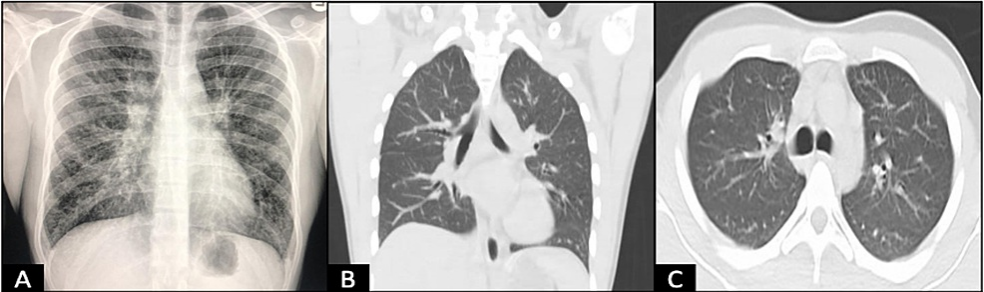
Radiographic findings of allergic bronchopulmonary aspergillosis (A) Posteroanterior (PA) chest radiography demonstrating reticulonodular interstitial markings; (B) coronal and (C) cross-section HRCT chest scans obtained one month after corticosteroid treatment. HRCT: high-resolution computed tomography

The initial chest radiography (CXR) scan was positive for reticulonodular interstitial lung markings (Figure 1A). Subsequent high-resolution computed tomography (HRCT) scan of the chest documented central bronchiectasis (CB) and extensive ground-glass opacities suggestive of interstitial lung disease (ILD). Of note, these findings were appreciated on official radiographical documentation, however, the radiographical images were not available for review. Laboratory workup were remarkable for *A. fumigatus* immunoglobulin G (IgG) level of 20.6 µg/mL (normal < 2 µg/mL), total serum IgE of 7001 kU/L (normal < 100 kU/L), and eosinophilia of 14,300 cells/µL (66.2%). Results from imaging and laboratory studies confirmed the diagnosis of ABPA with central bronchiectasis (ABPA-CB). 

**Figure 2:**
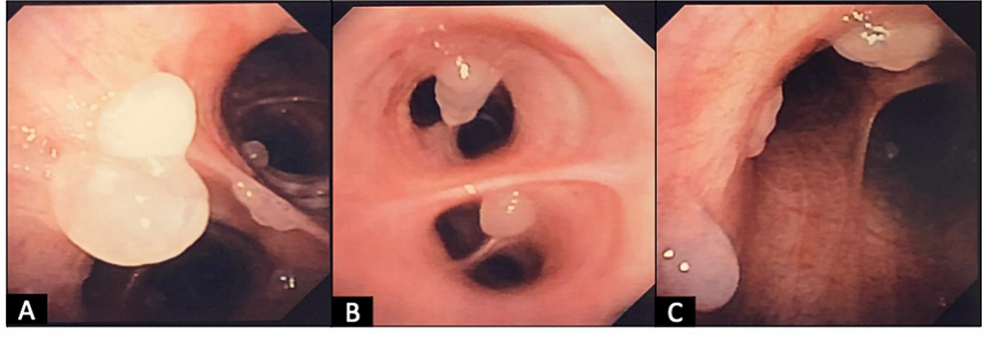
Findings of flexible fiberoptic bronchoscopy The presence of diffuse, non-obstructing mucus plugs (A) proximal (carina) and (B, C) distal airways after one month of corticosteroid therapy.

Treatment with oral prednisone 30 mg (0.5 mg/kg) daily for two weeks was commenced with the goal of alternating days of therapy for two months then followed by prednisone 5 mg daily taper every two weeks, as well as inhaled corticosteroids and albuterol rescue for symptomatic relief. Following one month of therapy, complete pulmonary function tests (PFTs) demonstrated significant improvement in airway obstruction and inflammation with an FVC of 67%, FEV1 of 72%, FEV1/FVC of 108%, post-bronchodilator FEV1 of 2% predicted values and FeNO of 38 ppm. Follow-up HRCT (Figure 1B, 1C) scan at this time showed a significant reduction of central bronchiectasis and ILD pattern. Total serum IgE had a 75% reduction to 1694 kU/L. A flexible fiberoptic bronchoscopy with bronchoalveolar lavage (BAL) was performed and revealed bilateral diffuse, non-obstructing mucinous plugs at proximal and distal airways (Figure 2A-2C). BAL fluid galactomannan (GM) index was 0.12 (normal 0.0 - 0.49) and ß-1,3-D-Glucan was 31 pg/mL (normal < 60 pg/mL). 

## Discussion

The pathogenesis of ABPA starts with inhalation of *A. fumigatus* spores with subsequent lodging on distal airways. Here, a localized immune response involving type I, III, and IV reactions as well as T helper 2 response that stimulates the production of interleukin-4, resulting in IgE isotype switching and eosinophil proliferation. This localized inflammatory response damages the bronchial epithelium and reduces the mucociliary capacity. This, in turn, causes accumulation of mucus and formation of mucus plugs around the airways. Plugged and inflamed airways ultimately lead to the development of bronchiectasis [10]. The thick, golden-brown mucus expectorated, as the one seen in our case, is typical of ABPA and contains eosinophils, desquamated epithelial cells, and mucin [1].

*Aspergillus* species are able to produce a wide range of pulmonary diseases ranging from aspergilloma within pulmonary cavitary lesions, ABPA, and invasive pulmonary aspergillosis (IPA). Disease manifestation typically depends on the host sensitization to the *A. fumigatus* as well as their current immune status. Specifically, ABPA is seen in immunocompetent hosts who have AS [11]. Since the prevalence of AS in asthmatic patients can be as high as 62%, they are considered to be predisposed to ABPA and, therefore, are included as part of most diagnostic criteria [12].

Nonetheless, diagnosing ABPA is particularly challenging given that symptoms tend to overlap with other more common pulmonary diseases and lack a gold-standard diagnostic criterion. The latest globally implemented diagnostic criteria were proposed by the International Society for Human and Animal Mycology (ISHAM) Working Group in 2013. Their criteria include (1) history of asthma or CF; (2) positive *Aspergillus* skin testing with a wheal diameter greater than 3 mm or elevated IgE against *A. fumigatus* above 0.1 kUA/L, and elevated total serum IgE above 1000 IU/mL; (3) at least two of the following: (a) precipitating serum antibodies against *A. fumigatus* or elevated serum *Aspergillus* IgG, (b) mucous plugging or central bronchiectasis seen on chest imaging, or (c) total eosinophil count greater than 500 cells/mL in steroid-naïve patients [13]. 

Even though our patient had bronchial asthma confirmed by spirometry upon our initial evaluation, the diagnosis of ABPA was most likely not considered because of his previous unremarkable past medical history. Upon review, multiple similar case reports remarked a delay in diagnosing ABPA due to the absence of prior asthmatic history [4, 5, 14]. In such cases, it cannot be determined if the patient never presented with asthmatic symptoms because they truly lacked bronchial asthma, did not recognize and failed to report their symptoms of fatigue, were never evaluated for the disease, or any combination of these reasons. Moreover, sputum production along with constitutional symptoms diverts the attention from asthma with ABPA and instead leads physicians to attribute symptoms to recurrent pneumonia or tuberculosis [15]. This case had the limitation of the patient presenting without previous spirometry tests in order to confirm or deny the presence of obstructive lung disease, such as asthma, prior to the onset of ABPA. However, a lack of asthma diagnosis or its symptoms should not rule out the possibility of ABPA [4]. In light of this, new 10-component diagnostic criteria were developed by Asano et al. [8] in Japan. Their new criteria were designed specifically for patients without a history of CF and are able to confirm the diagnosis of ABPA when at least six of the 10 components are met. These criteria were found to be more sensitive and specific when compared to the current diagnostic criteria being used.

Two radiologic hallmarks have been identified on HRCT scans of ABPA patients. The most prevalent is CB (76.5%), otherwise described as bronchiectasis that involves the proximal (hilar) bronchi. The second most common (20.9%) is high-attenuation mucus (HAM), which are mucus plugs that appear denser than the skeletal muscle tissue on CT. The presence or absence of these findings can further subclassify the diagnosis of ABPA into ABPA-CB or ABPA-HAM. If the patient lacks these radiological findings but has a confirmed diagnosis by serological studies, then the diagnosis is serological ABPA (ABPA-S). These classifications have been found to correlate with clinical severity and remission status. Patients with ABPA-CB are less likely to achieve remission status and those with ABPA-HAM suffer from recurrent relapses as compared with ABPA-S [16]. Remission is recognized when the patient has remained asymptomatic and with stably low IgE levels for six months after terminating corticosteroid and antifungal therapy. On the other hand, an increase in total serum IgE would represent a relapse of the disease [1].

Despite not being a requisite for establishing the diagnosis of ABPA with most diagnostic criteria, bronchoscopy with BAL was performed in our patient in order to identify or rule out other possible comorbidities such as IPA or other pulmonary infections. On bronchoscopy, bilateral, diffuse, non-obstructing mucus plugs were appreciated, comprising another one of the pathognomonic characteristics of ABPA [13]. BAL did not recover and grow other infectious organisms and other fungal markers such as GM index and ß-1,3-Galactomannan were within normal parameters. Importantly, the presence of *A. fumigatus *or positive GM index on BAL fluid cannot be used to differentiate between ABPA or colonization by itself [1, 17].

The treatment regimen was implemented as per established guidelines [1, 18]. In a recent randomized control trial, Agarwal et. al [19] established the superiority of oral glucocorticoids monotherapy in producing a response to treatment over Itraconazole monotherapy (100% vs 88%) in asthmatic patients with acute ABPA. Even so, itraconazole was still significantly effective and had the advantage of a safer side-effect profile as compared with glucocorticoids. For this reason, azoles should be added to the treatment regimen when the patient either cannot tolerate oral glucocorticoids or has not seen an adequate response to therapy [19]. 

Following one month of therapy, HRCT was remarkable for a lack of previously documented lesions of CB and ground-glass opacities. Similarly, PFTs showed significant improvement in the obstructive airflow pattern. However, the new pattern is concerning for restrictive lung disease. This is likely due to the nature of ABPA to develop everlasting lung injuries when the disease is manifested for a prolonged period of time. This further highlights the importance of early recognition and treatment of ABPA. 

## Conclusions

Herein we present the case of a previously healthy 21-year-old male who went undiagnosed and untreated for ABPA due to the lack of asthma per history. Delays in diagnosis and initiation of treatment have detrimental consequences for the patient’s health such as irreversible structural lung injuries and permanent loss of lung function, as seen in this case. Consequentially, given the good prognosis of adequately treated ABPA, we recommend that it is important that physicians should have a high suspicion for the disease when a patient presents as either difficult-to-treat asthma, drug-resistant pneumonia, or tuberculosis, regardless of their age or past medical history.
